# The Effects of Long-term Abacus Training on Topological Properties of Brain Functional Networks

**DOI:** 10.1038/s41598-017-08955-2

**Published:** 2017-08-18

**Authors:** Jian Weng, Ye Xie, Chunjie Wang, Feiyan Chen

**Affiliations:** Bio-X Laboratory, Department of Physics, Zhejiang University, Hangzhou, China

## Abstract

Previous studies in the field of abacus-based mental calculation (AMC) training have shown that this training has the potential to enhance a wide variety of cognitive abilities. It can also generate specific changes in brain structure and function. However, there is lack of studies investigating the impact of AMC training on the characteristics of brain networks. In this study, utilizing graph-based network analysis, we compared topological properties of brain functional networks between an AMC group and a matched control group. Relative to the control group, the AMC group exhibited higher nodal degrees in bilateral calcarine sulcus and increased local efficiency in bilateral superior occipital gyrus and right cuneus. The AMC group also showed higher nodal local efficiency in right fusiform gyrus, which was associated with better math ability. However, no relationship was significant in the control group. These findings provide evidence that long-term AMC training may improve information processing efficiency in visual-spatial related regions, which extend our understanding of training plasticity at the brain network level.

## Introduction

As is well known, training can result in improvements in various cognitive skills^1^. But the mechanisms underlying these cognitive gains remain unclear. In the past decade, neuroimaging researchers have focused on the training-induced plasticity and reorganization of neural systems^2, 3^, which may contribute greatly to our understanding of how training drives cognitive developments.

More recently, abacus-based mental calculation (AMC) training, a method to perform complex calculations in a visual-spatial format, has received much attention in the field of training-induced neural plasticity. Abacus, which consists of beads and rods, can be used to perform complex arithmetic operations, such as addition, subtraction, multiplication, division, square root and cubic root. Instead of a physical abacus, individuals after long-term AMC training can imagine an abacus in mind and perform arithmetic operations on a virtual abacus rapidly and precisely. For example, Stigler *et al*., found that AMC experts have extraordinary ability to do calculations involving multi-operands each with as many as 10 digits with unusual speed and high accuracy^4^. Recently, neuroimaging approaches have been used to study brain functional and anatomical plasticity induced by AMC training^2, 3, 5^. In fMRI studies with calculation tasks, AMC experts showed enhanced activation in some brain regions compared to the matched controls, possibly due to the strengthened involvement of a specific visual-spatial strategy, and the weakened involvement of the common linguistic strategy in AMC experts^2, 5^. These enhanced activations were often observed in brain regions including fusiform gyrus, inferior parietal lobule and occipital gyrus^2, 6, 7^. It has been proposed that the utilization of a visual-spatial representation in mathematical tasks may account for this phenomenon in AMC experts^8–11^. Anatomical MRI studies also indicated that frequent employment of visual-spatial processing in AMC training might result in structural plasticity^3, 12^. A voxel-based morphometric study provided evidence of between-group differences in the gray matter of AMC experts and controls^12^. Gray matter volume decreased in AMC group in visual-spatial related regions, such as fusiform gyrus^12^. Another study showed increased fractional anisotropy values in the occipitotemporal junction, a key fiber connected to fusiform gyrus and parietal lobule, which plays an important role in visual information processing^3^. Moreover, it was reported that the projections from the fusiform gyrus to other brain regions in AMC experts were strengthened in comparison with the control individuals^12^. Taken together, these functional and structural studies indicated that AMC training would result in different activation patterns and/or anatomical features in visual-spatial related regions, especially in the fusiform gyrus^2, 3, 12^, a key neural underpins of visual-spatial processing^13^. However, previous studies were based on voxel-based comparisons or seed-based connectivity, which both are less able to offer a complete view of the characteristics of whole brain connectivity. The present study, utilizing graph-based network analysis, assessed connectivity in a brain-wide network^14^. We hypothesized that long-term AMC training might induce the plasticity of brain functional network, especially in visual-spatial related regions, such as fusiform gyrus.

Nowadays, studies have implied that the human brain was organized as a complex system^15, 16^. Graph-theoretical network analysis is the most prominent method to quantify brain network properties and describe the framework of brain network organization^17, 18^. Using network analysis, studies have assessed brain network based on both structural and functional properties^17, 19–21^, with brain regions as nodes and the strength of region-to-region correlations as edges. Results have shown that human brain is organized as a network to process information efficiently by balancing brain integration and segregation^22^. Meanwhile, a large number of studies reported the alterations of brain networks induced by brain maturation, atrophy, psychiatric disorders, and skill acquisition^14, 17, 19, 20, 23, 24^. These studies indicated that brain network analysis could provide a complete view of the characteristics of brain connectivity and additional information about connectivity in specific neural systems.

Based on these findings, we hypothesized that AMC training might affect the organization of human brain network represented by topological alterations, especially in brain regions related to visual-spatial function, such as fusiform gyrus. To test these hypothesis, we recruited 72 children with AMC experience and 72 matched control children without any knowledge of abacus. A math ability test was used in our study, which is the most sensitive cognitive improvements induced by AMC training. Utilizing graph theory, we constructed brain functional networks, based on resting state MRI data, for each participant respectively. Then we contrasted the topological properties between the two groups. To the best of our knowledge, this is the first study to investigate the effect of AMC training on brain plasticity at the network organization level.

## Results

### Math ability

For children’s math performance, independent sample t-tests showed that the AMC group (56.97 ± 8.01) had higher scores than the control group (48.16 ± 8.30, *t* (142) = 6.44; *p* < 0.05).

### Comparisons of global network properties

The area under the curves (AUCs) of each network properties in the defined threshold range (from 24.5% to 30% with an interval of 0.005) were calculated and compared between two groups^25^. For the global network properties, there was no significant difference between AMC and control group in normalized clustering coefficient (AMC(AUC): 0.099 ± 0.008; control(AUC): 0.099 ± 0.007) and normalized shortest path length (AMC(AUC): 0.060 ± 0.002; control(AUC): 0.060 ± 0.001) (*p*_corrected_ > 0.05). In both group, no significant correlations were found between these global network properties and math ability (*p* > 0.05).

### Comparisons of regional network properties

**Figure 1:**
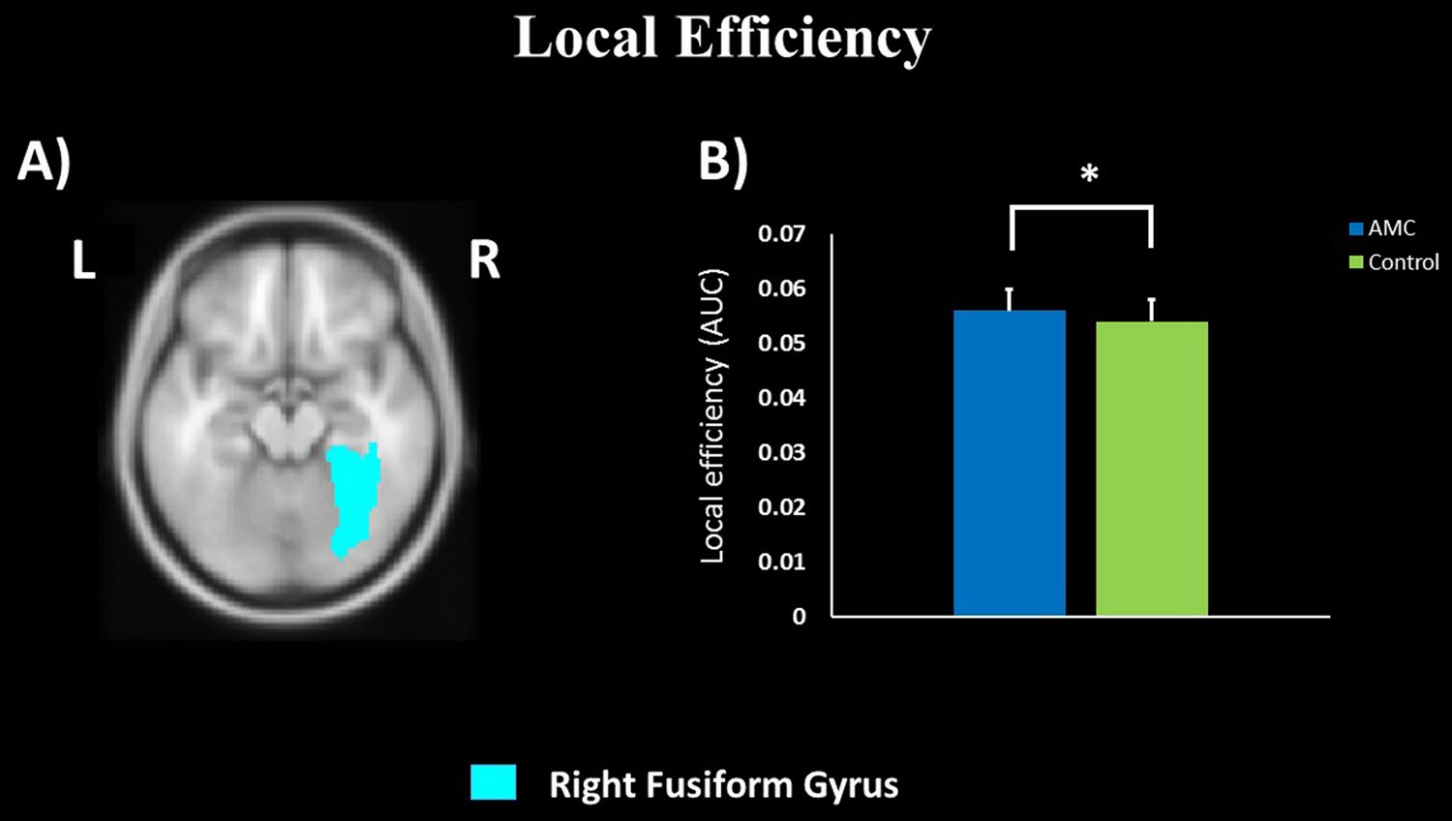
Group differences in nodal local efficiency in right fusiform gyrus. *p_corrected_ < 0.05. L: left, R: right.

**Figure 2:**
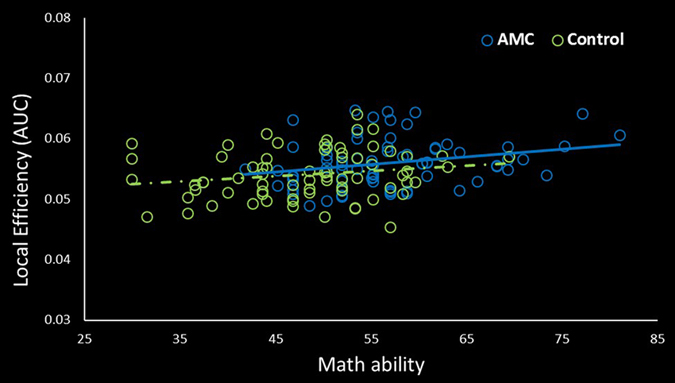
Relationship between math ability and local efficiency in right fusiform gyrus. In the AMC group, significant positive relationship was observed (*r* = 0.26, *p* = 0.03), while no correlation was found in the control group (*r* = 0.18, *p* > 0.05).

**Figure 3:**
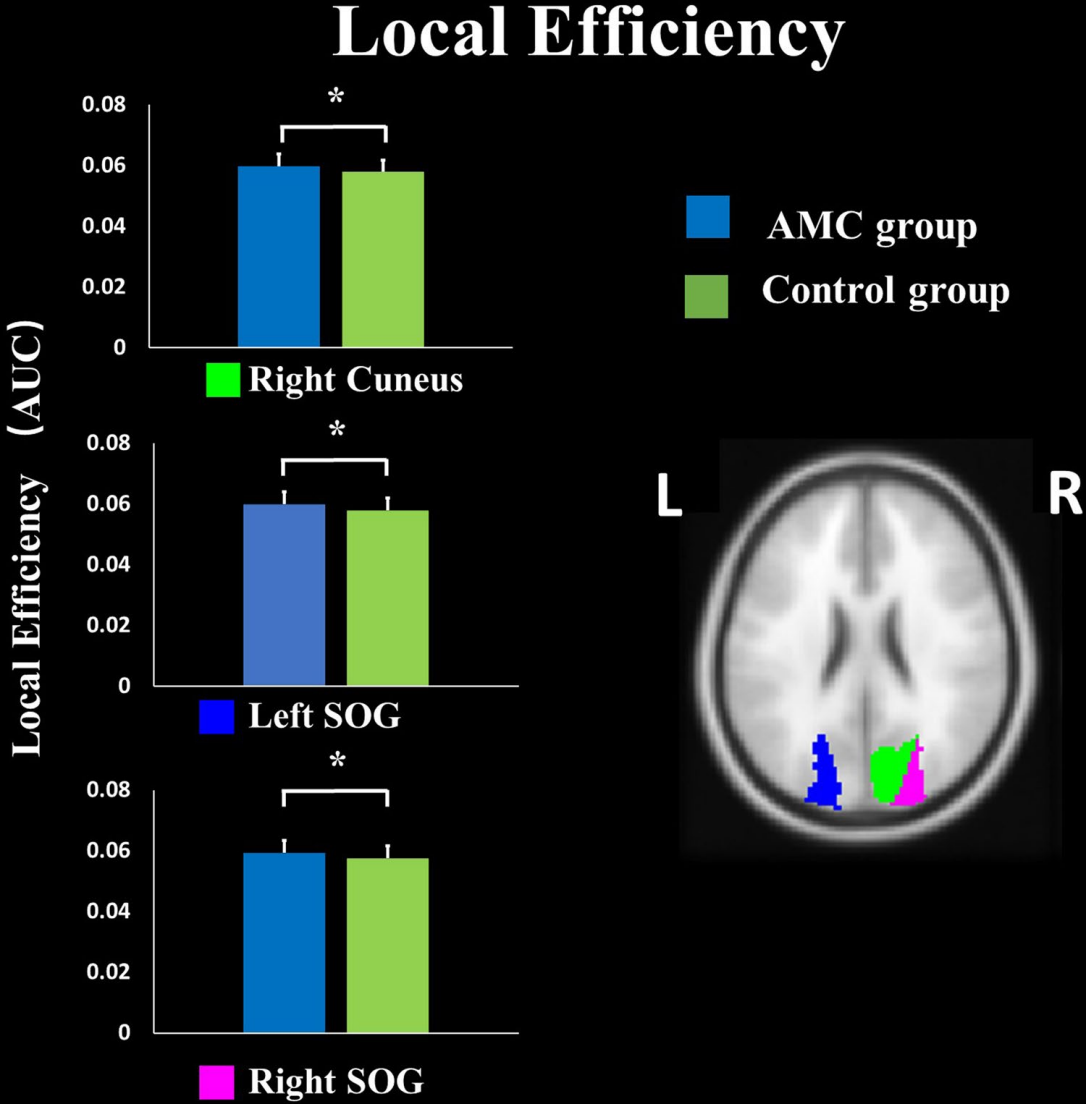
Group differences in local efficiency in bilateral SOG (superior occipital gyrus) and right cuneus. *p_corrected_ < 0.05. L: left, R: right.

**Figure 4:**
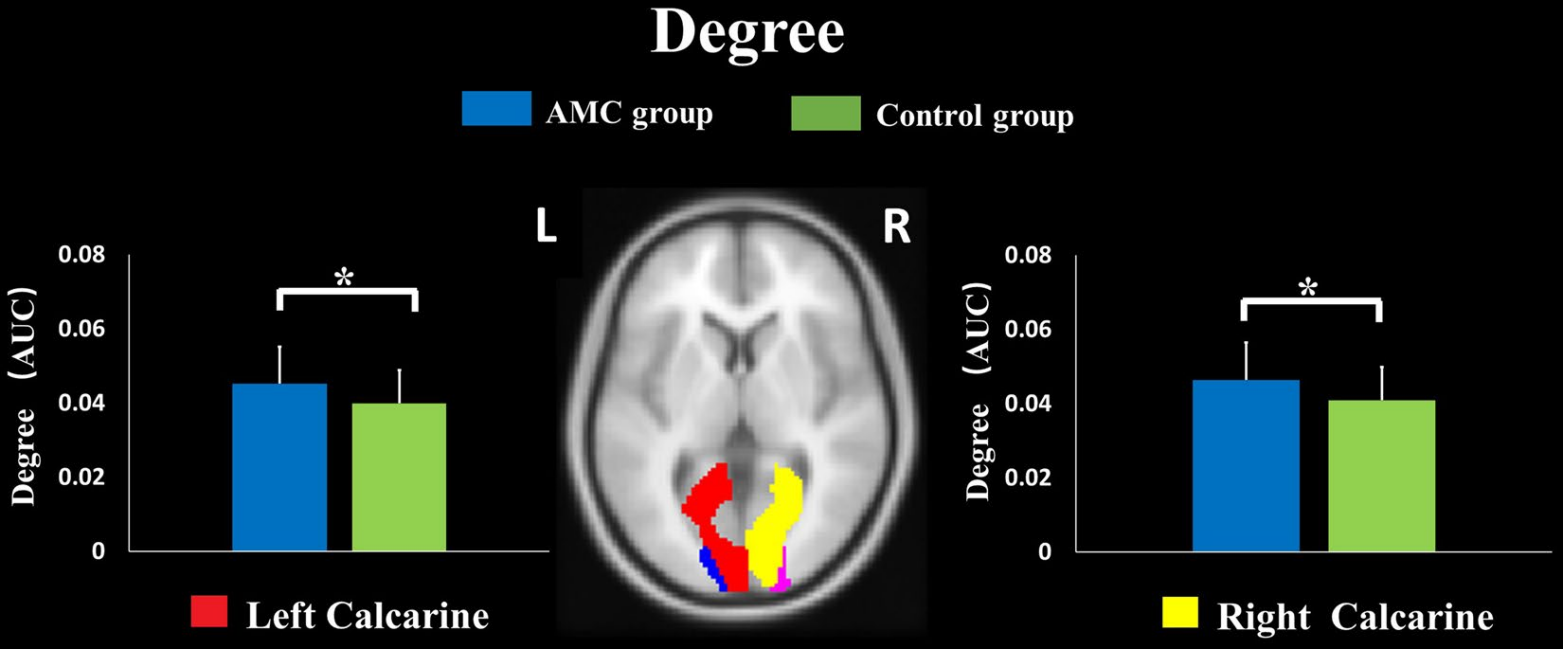
Group differences in nodal degree, bilateral calcarine sulcus. *p_corrected_ < 0.05. L: left, R: right.

**Figure 5:**
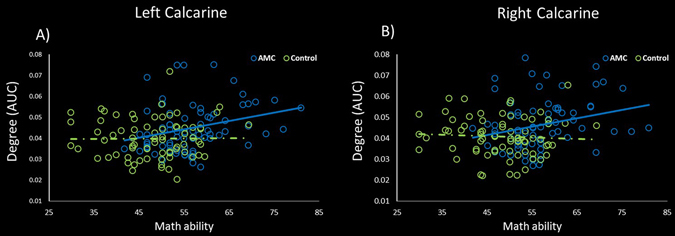
Relationship between math ability and nodal degree in bilateral calcarine sulcus. In the AMC group, significant positive relationship was observed (right calcarine sulcus: r = 0.27, *p* = 0.02; left calcarine sulcus: r = 0.27, *p* = 0.02), while no correlation was found in the control group (right calcarine sulcus: r = −0.06; left calcarine sulcus: r = 0.01; *p* > 0.05).

In comparison with the control group, the AMC group showed higher nodal local efficiency in right fusiform gyrus (*p*_corrected_ < 0.05, Fig. 1, Supplementary Fig. S1). The relationship between nodal local efficiency in right fusiform gyrus and math ability was also investigated. Positive correlation was found in the AMC group (*r* = 0.26, *p* = 0.03, Fig. 2), while no correlation was significant in the control group (*r* = 0.18, *p* > 0.05). Three other regions of AMC group also exhibited higher local efficiency (*p*_corrected_ < 0.05, Fig. 3, Supplementary Fig. S1), including bilateral superior occipital gyrus and right cuneus. We also examined the differences of the degree between the AMC and control groups. Higher nodal degrees in the brain areas of bilateral calcarine sulcus were found in the AMC group (*p*_corrected_ < 0.05, Fig. 4, Supplementary Fig. S2). Meanwhile, the nodal degree in bilateral calcarine sulcus was positively correlated with math ability in the AMC group (right calcarine sulcus: *r* = 0.27, *p* = 0.02; left calcarine sulcus: *r* = 0.27, *p* = 0.02, Fig. 5).

## Discussion

In the current study, we investigated the effects of AMC training on brain functional network organization. As to the behavioral performance, math ability was significantly higher in the AMC group than in the control group. At the neural level, we constructed brain networks for each participant and compared network properties between the AMC and control groups. Nodal-wise analysis showed higher degrees in bilateral calcarine sulcus and increased nodal local efficiency in bilateral superior occipital gyrus and right cuneus in the AMC group. More importantly, we found higher local efficiency of right fusiform gyrus in the AMC group. Additionally, local efficiency of right fusiform gyrus is positively correlated with math ability, which only occurred in AMC group. However, there was no significant between-group difference in normalized clustering coefficient and normalized mean shortest path length.

In line with our previous study^26^, AMC group performed better than their peers in the math test (*p* < 0.05). Previous studies indicated that individuals with AMC training would improve calculation speed and accuracy, and other mathematical performance^2, 5, 26, 27^. AMC experts have a tendency of the strengthened involvement of a specific visual-spatial strategy and the weakened involvement of the common linguistic strategy in numerical tasks^2, 5, 28^. A variety of behavioral studies have shown the influences of AMC training on promoting individuals’ numerical-related cognitive ability, such as digital memory span and calculation ability^8, 10, 11, 26^. In parallel, neuroimaging studies reported that there was more involvement of brain regions related to visual-spatial processing in mental calculation tasks for AMC experts than the controls, including the regions of parietal cortex, premotor areas and fusiform gyrus^2, 5^. On the contrary, general population employ a linguistic representation and rely on perisylvian language areas in arithmetic tasks^29^. In summary, studies provided the evidence that individuals with AMC expertise would widely use the visual-spatial strategy in numerical processing, and this would improve individuals’ numerical processing efficiency and mathematical performance^2, 5, 28^. Thus, there is no doubt that AMC experts in our study showed higher math ability.

In the current study, AMC experts exhibited higher nodal degree in bilateral calcarine sulcus relative to the control group (Fig. 4), and increased local efficiency in bilateral superior occipital gyrus and right cuneus (Fig. 3). All these regions are the major regions of visual processing center, receiving visual information and involving in visual information processing^30, 31^. Studies have reported the plasticity of visual cortex induced by AMC training^3, 28^. For example, Hu and his colleagues reported that AMC training could increase the fractional anisotropy values in a variety of junctions, such as the inferior longitudinal fasciculus (ILF), inferior fronto-occipital fasciculus (IFOF) and optic radiation (OR)^3^. These junctions, running from visual cortex to other regions, are mainly related to visual recent memory^32^ and transmission of visual signals to other brain regions^33^. Moreover, compared to control subjects, higher activation in superior occipital gyrus and cuneus have been reported in numerical working memory tasks for AMC experts^28^. An alternative reason is that individuals with expertise in AMC depend on brain areas involved in visual, visual-spatial, and visual-motor imagination in solving numerical problems^3, 5, 28^. In turn, long-term employing visual-related strategies to solve problems might induce plasticity of visual cortex^3, 12^. In our study, increase nodal degrees in bilateral calcarine sulcus, means more paths pass through these regions and more other regions connected with these regions. The establishments of connections might suggest that the importance of these regions in brain network has been improved; in other words, more information might be transferred and processed through these regions. We also found the math ability of AMC group was positively associated with nodal degree in bilateral calcarine sulcus (Fig. 5), which provided further evidence for our suggestion. Higher degree in bilateral calcarine sulcus is related with better task performance, which means information processing would be more efficient^18, 20^. Meanwhile, AMC experts with increased local efficiency in bilateral superior occipital gyrus and right cuneus might indicate that AMC training would improve efficiency of information processing in these regions. Based on these results, we concluded that long-term AMC training might enhance the importance of visual-related regions in the brain functional network, and improve the capabilities of information transmission and processing in these regions.

Another key finding of our study is that the AMC group showed higher local efficiency in right fusiform gyrus (Fig. 1), a region important to AMC training. Our result might suggest that the right fusiform gyrus in AMC group had closer local connectedness and higher information transfer efficiency in the network. As is well known, fusiform gyrus involved in visual-spatial information processing, such as viewing physical objects, imaging objects or manipulating imagery objects^13, 34, 35^. Activations in fusiform gyrus have been found in the tasks of individuals recognizing and presenting imagined objects^13, 34, 36, 37^. In AMC studies, enhanced activity has been reported in fusiform gyrus when AMC experts solve arithmetic problems^2, 5^. Researchers suggested this enhanced activity was related to the visual-spatial strategy used of AMC experts in arithmetical tasks. Furthermore, when individuals convert visual information from a not preferred mode (untrained category) to a preferred mode (trained category), activations in right fusiform gyrus would also be increased^7, 38^. To AMC experts, they would automatically transform linguistic representation (digit or abacus) to visual mental representation (information stored on an imagined abacus as a visual style)^12^. These processing strategies might enhance the role of fusiform gyrus in solving numerical tasks for AMC experts than the controls. In turn, long-term AMC training would alter function and structure in fusiform gyrus^2, 3, 12^. Moreover, diffusion studies indicated that AMC training might strengthen the junctions connected between fusiform gyrus and other brain regions^3, 12^. Stated thus, we have reasons to believe that AMC training might promote the efficiency of information processing in right fusiform gyrus. Further evidence showed that math ability was positively correlated with local efficiency in right fusiform gyrus (Fig. 2). Long-term AMC training would improve math ability, by visual-spatial strategy widely employed in training procedure^26^. Therefore, it is reasonable that the scores of math test positively related with the efficiency of right fusiform gyrus. Taken together, both neuroimaging and behavioral results in this study provide evidence that AMC training might induce connectivity plasticity in fusiform gyrus.

The results showed no significant between-group differences in normalized clustering coefficient and normalized shortest path length. These two parameters were proposed to measure information processing capability of the whole network. A possible interpretation for these results is that AMC training is a strategy-based training and mostly affects the regions related to the training-related strategy. We speculated that alterations of local properties induced by AMC training might be more sensitive than those of properties which measure the global network characters^39^.

It should be acknowledged the limitations in our study. Firstly, the ROIs based on the AAL atlas were used, however, different size of ROIs would introduce variations in graph-theoretical network analysis^40^. In the future work, more elaborated ROIs definition should be compared. Secondly, the correlation results between network properties and math ability could not survive the FDR corrections. In the present study, nodal network properties were computed in whole brain resting-state network, which might introduce variations to investigate the correlation between properties and a specific cognition (math ability). It might be better to explore relations between specific cognitions and topological properties of cognition-related networks in our future research. Finally, although a control group with high math ability, which could match to the AMC group, was hard to recruit, it will leave us a large space to improving the design of control group.

## Conclusions

In conclusion, the current study adopted graph-based network analysis to investigate the plasticity of functional networks in AMC experts. Our results suggested that long-term AMC training might contribute to individual’s math ability and induce functional network plasticity in visual-spatial related regions, such as right fusiform gyrus. These AMC-training-induced topological alterations might mainly relate to visual-spatial strategy employed widely in the training. Previous studies demonstrated that AMC training would change gray matter volume, the diffusion parameter of white matter and activation patterns in cognitive tasks^2, 3, 12^. This study provides evidence for the plasticity of brain functional network induced by long-term AMC training, and helps us to understand deeply how the brain changes induced by long-term training or learning.

## Methods

### Participants

In this study, one hundred forty-four children were recruited from a single city in China. Seven-two participants with at least one-year AMC training constituted the AMC group (9.56 ± 1.67 years, 33 males), and the others without any abacus or AMC knowledge constituted the control group (9.87 ± 1.63 years, 32 males). The AMC group was able to get rid of physical abacus and perform calculation by operating an imaginary abacus in mind. The Chinese version of Combined Raven test was employed in our study^26, 41^, so as to compare whether the two groups were matched on intelligence. No significant difference was detected between the AMC (mean ± SD: 107.94 ± 12.40) and control groups (mean ± SD: 104.50 ± 13.65) (*t* (142) = 1.57; *p = *0.11). The study protocol was approved by the ethics research committee of Zhejiang University in China and performed in accordance with the approved guidelines and regulations. Informed consent was obtained from all subjects and their parents. All subjects had no reports of any neurological or psychiatric disorders.

### Math Test

In the current study, math ability was measured by the Chinese version of the Heidelberger Rechentest (CHRT)^26, 42, 43^. Practice trials were provided before the formal testing to help children understand the task rules. A composite t score was obtained for each child according to the Chinese adapted city norm.

### Data Acquisition and Preprocessing

MRI data were acquired on a 1.5-T MRI clinical scanner (Achieva, Philips) with an 8-channel head coil in the First Hospital of Qiqihar. The resting state images were collected using a single-shot gradient-echo echo-planar imaging (EPI) sequence with the following parameters: TR/TE = 2000/50 ms, flip angle = 90°, FOV = 230 mm × 230 mm, matrix = 64 × 64, slice thickness/gap = 5 mm/0.8 mm, and 22 slices in an interleaved ascending order to cover the whole brain. These images were set obliquely and parallel to the anterior and posterior commissure line. The entire resting state scan included 180 scans and lasted for 360 s. Another high resolution anatomical scan was also obtained for each participant using a 3D fast field echo (FFE) sequence with the following parameters: TR/TE = 25/4.6 ms, flip angle = 15°, FOV = 256mm × 256 mm, acquisition matrix = 256 × 256, reconstruction voxel size = 1 × 1 × 1 mm^3^, 150 slices in the sagittal plane.

Image data were analyzed using SPM8 (http://www.fil.ion.ucl.ac.uk/spm) on Matlab (version 2010b; The MathWorks, Natick, MA, USA). The first five scans were discarded to remove the impact of magnetization stabilization. Data were subsequently corrected for slice timing differences. Functional image volumes were then registered and aligned to the first time-point image using a rigid-body transformation to correct head motion. Participants with a single displacement in any direction greater than 2 mm (translational movements) or 2° (rotational movements) were excluded prior to further analyses and not included in the final samples. For our participants were all children, Diffeomorphic Anatomical Registration Exponentiated Lie algebra (DARTEL) was applied to create a study-specific anatomic template across all subjects to optimize interparticipant registration^44^. Data were then smoothed with a 6-mm full-width half maximum (FWHM) Gaussian kernel, filtered with a band pass (0.01 ~ 0.08 Hz) to minimize the effects of cardiac and respiratory fluctuations, and removed the linear trend. Finally, several nuisance variables, including the 6 rigid body motion parameters and average white matter (WM), cerebrospinal fluid (CSF), and global time-series were removed by multiple linear regression analysis.

### Network construction

For functional connectivity analysis, 90 regions based on AAL template were defined as network nodes, and average time courses from these regions were extracted. Each participant’s correlation matrix was constructed respectively based on pair-wise Pearson correlation between the time courses of all brain regions. The diagonal elements of the constructed matrix were set to zero to remove correlations between a region and itself. Next, a network threshold, representing the fraction of present connections to the all connections, was defined to remove weak connections based on the value of correlation coefficient. This would generate a binary adjacency matrix, as the binary undirected brain network. In order to minimum variations introduced by selecting a single threshold, we set a threshold range^25, 45^. The minimum threshold was set at 24.5%. Previous studies indicated that human cognition was related to integration of different brain regions, and brain regions were not isolated in whole brain network^20, 46^. We found that at the minimum threshold of 24.5%, all participants’ network was a full-connected network and each brain region was at least connected to another one in the network. To remove weak correlations, the P-values of correlation matrix were corrected using the false discovery rate correction (q = 0.05). Applying this procedure to each participant resulted in a threshold at 30.19% so that all remained correlations were significantly strong, and we selected the integer (30%) as the maximum density in the current study. Larger than this threshold the graph became random and the network would be less likely biological for brain functional network^47^. Thus, the final threshold range was from 24.5% to 30% with an interval of 0.005. At each threshold, network properties were calculated.

### Global network analyses

The clustering coefficient *C* and the characteristic shortest path length *L* were calculated to reflect the global topological properties of brain network in this study. The *C* is the mean of the local clustering coefficients of all the nodes, a measure of the number of edges between its nearest neighbors, and represents network segregation. The *L* of a graph is the mean of shortest path lengths between all pairs of nodes in the network and is the most commonly used measurement of network integration^48^. Comparing to 1000 matched random networks, the normalized shortest path length (*L*_nor_) and normalized clustering coefficient (*C*_nor_) were calculated from *L* and *C* to characterize the topology of brain networks^19^.

### Regional network analyses

Two nodal characteristics of degree and local efficiency were computed for individual’s network respectively and compared between two groups. The degree of a node is the number of edges connected to the node, and it measures the interaction of a node with the whole network^20^. Local efficiency is the inverse of the average shortest path length between all immediate neighbors of a node, as a measure of the efficiency of local information transfer between neighboring nodes^19^.

### Statistical Analysis

The AUCs of topological properties were calculated for statistical analysis^25^, with the threshold ranging from 24.5% to 30% with an interval of 0.005. The AUC methods would decrease the variations induced by threshold selecting and is sensitive to detect network alterations^25, 45^. Between-group differences of topological properties were examined by a non-parametric permutation test with 10000 repetitions^49^. In each repetition, the data of subjects were randomly assigned into two randomized groups, corresponding to the two groups respectively. The between-group differences of network topological properties between randomized groups were computed and formed the permutation distribution. Finally, a two-tailed *p*-value was computed based on the percentile position of the place where the real training-induced difference was in the permutation distribution. The false discovery rate (FDR, q = 0.05) methods were employed for multiple correction.

For those nodes showing significant between-group differences, Pearson correlation analyses were carried out to test the relationships between behavioral performance and network properties. Significant correlation threshold was set at *p* < 0.05.

### Data availability statement

The datasets generated in the current study are available from the corresponding author on reasonable request. Interested researchers could contact Dr. Feiyan Chen by e-mail chenfy@zju.edu.cn.

## Electronic supplementary material

supplementary figure
