# Supplementary material for: The Effects of Long-term Abacus Training on Topological Properties of Brain Functional Networks
Source: Sci Rep. 2017 Aug 18;7:8862. doi: 10.1038/s41598-017-08955-2 (PMC5562922; doi:10.1038/s41598-017-08955-2)
Supplement: Supplementary file 1 — supplementary figure [file 41598_2017_8955_MOESM1_ESM.pdf]

The Effects of Long-term Abacus Training on Topological Properties of Brain Functional Networks

Jian Weng[1], Ye Xie[1], Chunjie Wang[1], Feiyan Chen[1*]

[1] Bio-X Laboratory, Department of Physics, Zhejiang University, Hangzhou, China

[*] Correspondence to: Feiyan Chen, Bio-X Laboratory, Department of Physics, Zhejiang University, 38 Zheda Road, Hangzhou 310027, China.

E-mail: chenfy@zju.edu.cn

Fax:+86-571-8795-3997.

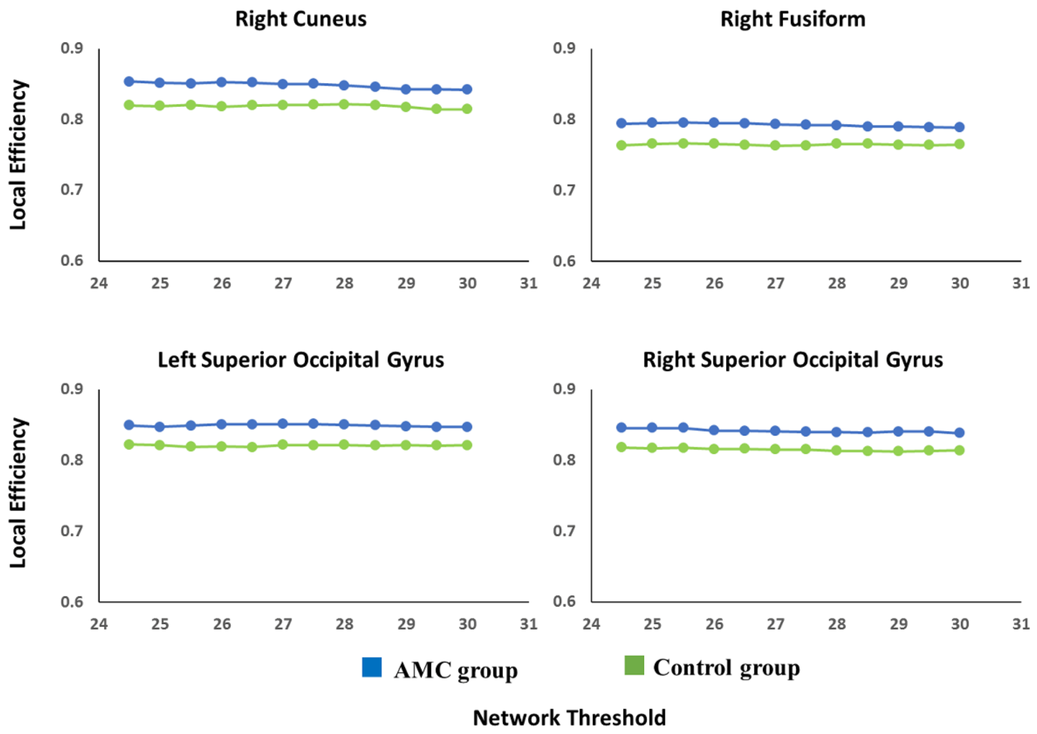

Supplementary Fig. S1. The curves of local efficiency in the right cuneus, fusiform and bilateral superior occipital gyrus from the minimum density (24.5%) to maximum density (30%) for both groups.

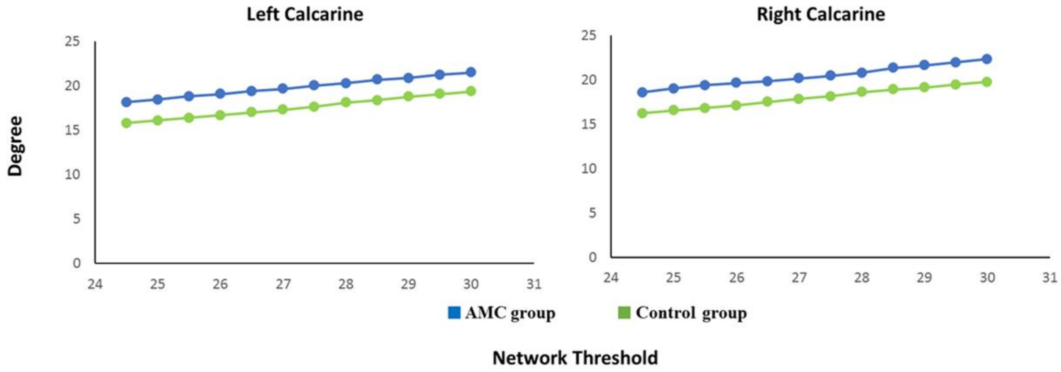

Supplementary Fig. S2. The curves of degree in bilateral calcarine from the minimum density (24.5%) to maximum density (30%) for both groups.
